# Evaluation of an HPV16-L1 antibody rapid test for oropharyngeal cancer diagnosis: diagnostic accuracy and challenges in real-world settings

**DOI:** 10.1016/j.ebiom.2025.106057

**Published:** 2025-12-11

**Authors:** Johannes M.A. Kusters, Nicole Brenner, Johannes A. Bogaards, Steffi Silling, Mariam El-Zein, Penelope Gray, Hanna Kann, Eduardo L. Franco, Matti Lehtinen, Andreas Dietz, Simon Laban, Jens Peter Klussmann, Ulrike Wieland, Tim Waterboer

**Affiliations:** a Infections and Cancer Epidemiology Division, German Cancer Research Center (DKFZ), Heidelberg, Germany; b Department of Epidemiology and Data Science, Amsterdam UMC, Amsterdam, the Netherlands; c Institute of Virology, National Reference Center for Papilloma- and Polyomaviruses, University of Cologne, Faculty of Medicine and University Hospital Cologne, Cologne, Germany; d Division of Cancer Epidemiology, McGill University, Montreal, QC, Canada; e Center for Cervical Cancer Elimination, Department of Clinical Science Intervention & Technology, Karolinska Institutet, Stockholm, Sweden; f Department of Microbiology and Immunology, University of Gothenburg, Gothenburg, Sweden; g Department of Otolaryngology, Head & Neck Surgery, University Hospital of Leipzig; h Department of Otorhinolaryngology and Head & Neck Surgery, University Hospital of Ulm, Ulm, Germany; i Department of Otorhinolaryngology, Head and Neck Surgery, Medical Faculty, University of Cologne, Cologne, Germany; j Center for Molecular Medicine Cologne (CMMC), University of Cologne, Faculty of Medicine and University Hospital Cologne, Cologne, Germany

**Keywords:** Human papillomavirus, Oropharyngeal cancer, Screening, Antibodies, Vaccination, Infection

## Abstract

**Background:**

Diagnostic assays have been introduced to diagnose human papillomavirus (HPV)-driven oropharyngeal cancer (HPV-OPC), including those identifying HPV16-L1 antibodies. This study aims to evaluate the diagnostic accuracy of an HPV16-L1 antibody rapid test for HPV-OPC, and its performance in individuals likely to have HPV16-L1 antibodies from causes other than HPV-OPC.

**Methods:**

Serum samples (n = 235) from three study populations were tested using a CE-certified serological HPV16-L1 antibody rapid test (Prevo-Check®) at the German National Reference Center for Papillomaviruses. Laboratory personnel were blinded to participant characteristics and followed the manufacturer's instructions. The three study populations consisted of: (1) patients with HPV16-positive or -negative OPC (n = 83), (2) bivalent (HPV16/18) vaccine recipients (n = 50), with paired baseline and one-month post-third-dose serum samples, and (3) naturally HPV16 infected young adults (n = 26), with paired serum samples before and after HPV16 seroconversion.

**Findings:**

In the study population with patients with OPC, the sensitivity of the HPV16-L1 antibody rapid test to detect HPV-OPC was 25.0% (95% CI: 13.6, 39.6), and its specificity was 97.1% (95% CI: 85.1, 99.9). The positive predictive value was 92.3% (95% CI: 64.0, 99.8) and negative predictive value 48.6% (95% CI: 36.4, 60.8). In the other study populations, the test was negative for all pre-vaccination samples, and all samples collected before incident natural HPV16 infection. Nearly all post-vaccination samples (98.0%), and one-third of the samples after natural HPV16 infection (34.6%) tested positive in the HPV16-L1 antibody rapid test.

**Interpretation:**

The HPV16-L1 antibody test has low diagnostic accuracy and cannot reliably distinguish different sources of HPV16-L1 antibodies. Therefore, this type of assays is not suitable for screening and detection of HPV16-driven OPC.

**Funding:**

This study is supported by the Ministry of Research, Technology and Space (BMFTR) core bvfunding provided to DKFZ. The NRC for Papilloma- and Polyomaviruses is supported by the Ministry of Health (BMG, grant no. 1369-401).

**Research in context**

**Evidence before this study**

HPV is the most common sexually transmitted infection and has the potential to cause cancers, including cervical and oropharyngeal cancer. HPV-driven oropharyngeal cancer has been rising in recent decades, and early detection is difficult because there are no known precursor lesions. Blood-based rapid tests detecting HPV16-L1 antibodies have been suggested as minimally invasive tools for identifying HPV-driven oropharyngeal cancer. Some studies showed promise in cancer patients, but it is still unclear how these tests perform in real-world settings, such as in individuals with HPV16-L1 antibodies from other causes, including recent vaccination or natural infection. In these cases, antibody positivity may reflect prior exposure rather than disease, limiting their usefulness for cancer screening or early detection.

**Added value of this study**

We evaluated a CE-certified HPV16-L1 antibody rapid test in three groups: patients with oropharyngeal cancer, individuals recently vaccinated against HPV, and those with recent natural HPV16 infection. The test identified only a quarter of HPV-driven cancers, while nearly all vaccinated individuals and about a third of naturally infected individuals also tested positive. This demonstrates both low sensitivity for detecting cancer and the potential for false positives in non-cancer populations, highlighting limitations of this test for diagnostic or screening purposes.

**Implications of all the available evidence**

HPV16-L1 rapid tests cannot reliably distinguish antibodies resulting from HPV vaccination, natural HPV infection, or HPV-driven oropharyngeal cancer. They are therefore unsuitable for population screening or early detection of HPV-driven oropharyngeal cancer. Future efforts should focus on identifying alternative biomarkers and understanding the progression from HPV infection to oropharyngeal cancer.

## Introduction

Human papillomaviruses (HPV) are small non-enveloped DNA viruses, and the most common sexually transmitted infection worldwide.^1^ There are more than 200 HPV genotypes, of which twelve are classified as high-risk HPV by the World Health Organisation, due to their potential to cause cancer.^2^ The most common HPV-driven cancer is cervical cancer, with an estimated 570,000 annual cases globally.^3^ The second most frequent HPV-driven cancer is head and neck cancer, particularly oropharyngeal cancer (OPC) arising from the tonsils and the base of the tongue.^4^ It is estimated that globally over 42,000 HPV-driven OPC (HPV-OPC) cases occur annually,^3^ with a sharp increase in incidence observed over the last decades.^5^

Primary prevention of HPV-related cancers can be achieved through prophylactic vaccination against infection with oncogenic HPV genotypes. Recent studies have demonstrated that HPV vaccination is highly effective in preventing cervical cancer.^6^ However, its effectiveness in preventing HPV-OPC is yet uncertain, as the average age of HPV-OPC diagnosis is significantly higher than that of cervical cancer.^7^ Additionally, HPV-OPC predominantly affects men,^4^ while HPV vaccination campaigns have historically primarily targeted women, and the vaccination uptake in many countries has been suboptimal.^8^ As a result, the extent of primary prevention against HPV-OPC provided by prophylactic HPV vaccination may only become evident decades from now. Moreover, precursor lesions that could serve as a proxy for estimating HPV vaccine effectiveness in an earlier stage have not been idenified.^9^

Secondary prevention of cervical cancer focuses on screening for (pre-)cancerous lesions, which has led to a reduction in cervical cancer incidence.^10^ Screening and early detection of HPV-OPC is more challenging, due to the absence of known precursor lesions at this site.^9^ However, with increasing trends in HPV-OPC incidence, developing diagnostic tools for screening and early detection of new cases would be beneficial. Several assays have been introduced to detect HPV-OPC at an early stage and are therefore suggested for HPV-OPC screening, including blood-based rapid tests detecting HPV16-L1 antibodies.^11^^,^^12^ Minimally invasive blood-based assays have the advantage of not having to collect a tissue sample. Previously, promising results were reported of an HPV16-L1 antibody rapid test for the classification of HPV-OPC and as a clinical biomarker of disease recurrence after treatment.^11^ However, its use as a diagnostic or screening tool for HPV-OPC may be considered controversial in certain real-world settings. All currently available prophylactic HPV vaccines are based on virus-like-particles composed of HPV L1 capsid proteins which elicit high-titre L1 antibodies.^13^ Therefore, vaccinated individuals may test positive on HPV16-L1 antibody rapid tests, even in the absence of HPV-OPC. Additionally, multiple studies have shown that antibodies against HPV-L1 can result from natural HPV infections, while most of these infections are cleared and hence transient.^14^, ^15^, ^16^ Consequently, the presence of HPV-L1 antibodies would indicate HPV exposure, rather than disease.

This study evaluates the diagnostic accuracy of an HPV16-L1 rapid test as a diagnostic tool for HPV-OPC in both HPV-OPC and non-HPV-OPC patients. Furthermore, potential challenges in individuals without HPV-OPC are explored, including those recently vaccinated against HPV and those who have had a recent natural HPV16 infection.

## Methods

### Design

The study had two aims: (1) assessing the diagnostic accuracy of a CE-certified HPV16-L1 antibody rapid test (Prevo-Check®) as a diagnostic tool for the detection of HPV-OPC, and (2) evaluating the test's performance in individuals who may have HPV16 antibodies due to reasons other than HPV-OPC. To achieve this, the HPV16-L1 antibody rapid test was evaluated using serum samples from three different study populations: (1) patients with OPC (both patients with HPV-driven and non-HPV-driven OPC) to determine the rapid test's diagnostic accuracy, and for the second aim, (2) HPV vaccine recipients (bivalant HPV16/18 vaccine, Cervarix®) and (3) individuals with natural HPV16 infections, for the second aim.

### Study populations and samples

#### Study population 1: patients with OPC

Serum samples and tumour tissue were collected from female and male patients with OPC, diagnosed and treated at the University Clinics of Gießen, Germany between 2013 and 2017. Sex of the participants were retrieved from medical records. Participants were consectutively selected and samples were obtained at diagnosis and before treatment. HPV status of patients with OPC (n = 83) was defined by detection of HPV-DNA using PCR and reverse hybridisation with the RDB2270 HPV Easy-Typing kit, according to the manufacturer's recommendations (Autoimmun Diagnostika GmbH, Strassberg, Germany) and p16^INK4a^ immunohistochemical staining (E6H4clone; CINtec Plus; Roche Diagnostics, Mannheim, Germany), which is commonly used in clinical settings. OPC patients with a positive result for both p16 overexpression and HPV16 DNA were classified as having HPV-OPC (n = 48). Individuals were defined as HPV-negative OPC cases (n = 35), if they were negative for both HPV16 DNA and p16 overexpression (n = 32), HPV16 DNA negative with unknown p16 status (n = 2), or HPV16 DNA positive but with a negative p16 status (n = 1).

#### Study population 2: HPV vaccine recipients

Paired serum and genital swab samples were obtained (2004–2005) before and after complete vaccination with the bivalent HPV16/18 virus-like-particle (VLP) vaccine (Cervarix®, GSK) from 16 to 17 year-old Finnish self-reported females (n = 50) enrolled in a phase III vaccine trial.^17^ Samples were collected at baseline (i.e., month 0, before receiving the first dose of bivalent HPV vaccine) and one month after receiving the third vaccine dose (i.e., month 7). All vaccine recipients (n_participants_ = 50, n_samples_ = 100) had a negative genital HPV DNA test result and were HPV seronegative for all tested genotypes at baseline of the study.^17^^,^^18^ Therefore, participants were considered as being previously HPV-uninfected.

#### Study population 3: naturally HPV infected individuals

Paired serum samples and anogenital swab samples were collected (2006–2012) from participants of the longitudinal “HPV Infection and Transmission among Couples through Heterosexual activity” (HITCH) study conducted in Montreal, Canada.^19^ Females (aged 18–24 years) and their male partners (aged 19–26 years) provided self-collected vaginal or nurse-collected penis and scrotum swabs, respectively, for HPV DNA detection, along with blood samples. Sex in this study was self-reported. For the current analyses, participants were selected independently of their partner (i.e., couples were not selected together for the present study) based on having (1) an incident genital (i.e., vaginal, penile or scrotal): HPV16 DNA positive result, and (2) a subsequent positive HPV16-L1 antibody result, tested with multiplex serology (procedures below). For each participant, the last seronegative and first seropositive serial serum samples were included in the analysis (n_participants_ = 26; n_samples_ = 52). All participants were genital HPV DNA negative and HPV seronegative at baseline; therefore, they were considered previously uninfected with HPV.

### HPV16-L1 serological rapid testing

All serum samples were tested using a CE-certified HPV16-L1 antibody rapid test (Prevo-Check®, Abviris)^11^ on two consecutive days in November 2020 at the German National Reference Center (NRC) for Papilloma- and Polyomaviruses at the University of Cologne, Germany. The investigators and lab personnel at the NRC were blinded to the allocation of sera among the three study populations. The Prevo-Check® assay was performed in accordance with the manufacturer's instructions (Lot numbers 19112802.1 and 20030906.1). Briefly, 25 μl of serum (at room temperature) were pipetted into a vial containing the Prevo-Check® HPV reagent. The serum and reagent were mixed by pipetting up and down several times. With the lid closed, the tube was gently inverted and tapped on a solid surface to collect any remaining liquid from the lid. After 10 min at room temperature, 100 μl of the mixture were pipetted into the sample well of the test cassette, carefully avoiding air bubbles. After exactly 10 min, the test was visually evaluated according to the manufacturer's instructions, and the reaction field of the test cassette was photo-documented. The HPV16-L1 antibody rapid test provides a binary outcome (e.g., negative or positive result). A negative external control (serum from an HPV-naïve person) was measured at least once per lot and once per test day. Serum from a recently HPV-vaccinated (Gardasil9®) young adult served as a positive control.

### HPV16-L1 multiplex serology

In addition to the rapid test, serum samples were tested for HPV16-L1 antibodies with a widely used serological research assay (multiplex serology), as previously described.^20^ For study population 1, this testing was performed as part of the current study, while for the other two study populations, it had been performed as part of the original studies.^17^^,^^19^ In brief, the HPV16-L1 antigen was bacterially expressed as glutathione-S-transferase (GST)-L1-fusion proteins, and non-covalently loaded onto glutathione-casein coupled polystyrene beads (Luminex). In addition to HPV antigens, GST was loaded onto one additional bead set for background determination. Antigen-loaded beads were combined into one bead mix and presented to primary serum antibodies (final serum dilution 1:100). Formed immunocomplexes were detected using a triple-specific biotinylated goat-α-human-IgG/IgM/IgA secondary antibody and streptavidin-R-phycoerythrin as reporter dye. Antibody levels were measured in a Luminex 200 flow cytometer as median fluorescence intensities (MFI) for at least 100 beads per bead set and serum sample. The investigators and lab personnel were blinded to the allocation of sera among the three study populations. Seropositivity for HPV16-L1 was defined by the pre-specified cut-off of 422 MFI.^21^

### Statistical analyses

The characteristics of each study population were summarised using descriptive statistics. The results of the HPV16-L1 antibody rapid test were presented separately for the three study populations (i.e., patients with OPC, HPV vaccine recipients, and natural HPV infected individuals).

To assess the diagnostic accuracy of the HPV16-L1 antibody rapid test to detect HPV-OPC, the sensitivity, specificity, positive predictive value (PPV), negative predictive value (NPV), and correctly classified proportion were calculated using concomitant positivity for HPV16 DNA and p16 as the reference method.

Finally, for each study population, the results of the rapid test were compared to those of HPV16-L1 multiplex serology. Positivity for both outcomes (i.e., Prevo-Check and multiplex serology) were tested using McNemar's test, without continuity correction due to small sample sizes. A significant result indicates a significant difference in marginal positivity results between the two tests, suggesting that one test detects HPV-L1 positivity more frequently than the other test.

All analyses were conducted separately for each study population. Records with missing data for key variables were excluded from analyses. Graphical illustrations were created with R version 4.3.2. The results are reported according to the Standards for Reporting Diagnostic Accuracy Studies (STARD) 2015 guidelines.^22^

### Ethics

All study participants provided writen informed consent. The protocol from study population 1 was approved by the ethical review committee of the Justus-Liebig University of Gieβen (AZ.: 95/15), from study population 2 was approved by the Finnish National Ethical Review Board (ETENE/Tukija), and from study population 3 was approved by the ethical review committees of McGill University (IRB Study Number A09-M77-04 A), Concordia University, and the Université de Montréal.

### Role of funders

Funders had no role in the study design, data collection, data analyses, interpretation or writing of this report.

## Results

The study population consisted of 159 individuals from three study populations who provided a total of 235 blood samples. The characteristics of the three study populations are presented in Table 1. Among the patients with OPC (first study population), most cancers were located at the tonsils (48/83, 57.8%), followed by the base of the tongue (15/83, 18.1%). These patients were mostly males, aged 42–91 years. Based on p16 overexpression and HPV16 DNA positivity, 48 of 83 patients with OPC (57.8%) were classified as HPV-OPC.

**Table 1:** Characteristics from the study populations: oropharyngeal cancer patients, HPV vaccine recipients, and naturally HPV infected individuals.

Study population 1: OPC patients^a^	Non-HPV-OPC	HPV-OPC
	n (%) or median [IQR]	n (%) or median [IQR]
**Total**	35	48
**Sex**		
Female	6 (17.1)	14 (29.2)
Male	29 (82.9)	34 (70.8)
**Age**	60 [56, 67]	63 [58, 71]
**Tumour site**		
Base of tongue	4 (11.4)	11 (22.9)
Glossotonsillar sulcus	1 (2.9)	1 (2.1)
Side wall	8 (22.9)	3 (6.2)
Soft palate	1 (2.9)	0 (0.0)
Tonsils	15 (42.9)	33 (68.8)
Uvula	2 (5.7)	0 (0.0)
Vallecula	4 (11.4)	0 (0.0)
**UICC7 stage**		
Stage I	4 (11.4)	0 (0.0)
Stage II	2 (5.7)	2 (4.2)
Stage III	7 (20.0)	15 (31.2)
Stage IVa	13 (37.1)	22 (45.8)
Stage IVb	5 (14.3)	5 (10.4)
Stage IVc	4 (11.4)	4 (8.3)
**p16-status**		
Negative	33 (94.3)	0 (0.0)
Positive	0 (0.0)	48 (100.0)
Unknown	2 (5.7)	0 (0.0)
**HPV16 DNA status**		
Negative	34 (97.1)	0 (0.0)
Positive	1 (2.9)	48 (100.0)


Study population 2: HPV vaccine recipients^b^	Pre-vaccination	Post-vaccination
	n (%) or median [IQR]	n (%) or median [IQR]
**Totals (paired)**	50	50
**Sex**		
Female	50 (100.0)	50 (100.0)
Male	0 (0.0)	0 (0.0)
**Age**	16 [16, 17]	17 [17, 17]
**HPV16 L1 antibody status**		
Negative	50 (100.0)	0 (0.0)
Positive	0 (0.0)	50 (100.0)


Study population 3: Naturally HPV infected individuals^c^	Pre-seroconversion	Post-seroconversion
	n (%) or median [IQR]	n (%) or median [IQR]
**Total (paired)**	26	26
**Sex**		
Female	23 (88.5)	23 (88.5)
Male	3 (11.5)	3 (11.5)
**Age**	21 [19, 22]	22 [21, 23]
**HPV16 DNA status**		
Negative	3 (11.5)	7 (26.9)
Positive	23 (88.5)	19 (73.1)
**HPV16 L1 antibody status**		
Negative	26 (100.0)	0 (0.0)
Positive	0 (0.0)	26 (100.0)

Abbreviations: HPV-OPC, HPV-associated oropharyngeal cancer; IQR, interquartile range; non-HPV-OPC, non-HPV-associated oropharyngeal cancer.

a HPV-OPC positivity was based on concurrent HPV16 DNA and p16^INK4a^ positivity, and otherwise was considered non-HPV-OPC.

b Post-vaccination was after receiving 3 doses of Cervarix® and 1 month after receiving the third dose. All vaccinated 16- and 17-year-old females were negative for HPV DNA at baseline and 7 months later.

c Seroconversion is based on last HPV16-L1 antibody negative and first HPV16-L1 antibody positive serial sample result after natural HPV infection.

The second study population consisted of only females aged 16–17 years, who provided blood samples before vaccination and one month after receiving a third dose of Cervarix®. The third study population consisted of individuals who seroconverted following natural infection with HPV16. The majority of participants were female (23/26, 88.5%), and ages ranged from 18 to 26 years.

In the OPC study population, 12 of the 48 (25.0%) HPV-OPC patients tested positive for the HPV16-L1 antibody rapid test (Prevo-Check®), and 36 (75.0%) tested negative (Table 2). Of the non-HPV-OPC, this was 1 (2.9%) and 34 (97.1%) out of 35, respectively.

**Table 2:** HPV16-L1 antibody rapid test (Prevo-Check®) results for an oropharyngeal cancer cohort, HPV vaccination cohort and natural infection cohort.

Study population 1: OPC patients	HPV16-L1 antibody rapid test (Prevo-Check®)	
	Negative	Positive
**Non-HPV-OPC**^a^ n (%)	34 (97.1)	1 (2.9)
**HPV-OPC**^a^ n (%)	36 (75.0)	12 (25.0)


Study population 2: HPV vaccine recipients	HPV16-L1 antibody rapid test (Prevo-Check®)	
	Negative	Positive
**Pre-vaccination**^b^ n (%)	50 (100.0)	0 (0.0)
**Post-vaccination**^b^ n (%)	1 (2.0)	49 (98.0)


Study population 3: Naturally HPV infected individuals	HPV16-L1 antibody rapid test (Prevo-Check®)	
	Negative	Positive
**Pre-seroconversion**^c^ n (%)	26 (100.0)	0 (0.0)
**Post-seroconversion**^c^ n (%)	17 (65.4)	9 (34.6)

Abbreviations: HPV-OPC, HPV-associated oropharyngeal cancer; non-HPV-OPC, non-HPV-associated oropharyngeal cancer.

a HPV-OPC positivity was based on concurrent HPV16 DNA and p16^INK4a^ positivity, and otherwise was considered non-HPV-OPC.

b Post-vaccination was after receiving 3 doses of Cervarix® and 1 month after receiving the third dose. All vaccinated 16- and 17-year-old females were negative for HPV DNA at baseline and 7 months later.

c Seroconversion is based on last HPV16-L1 antibody negative and first HPV16-L1 antibody positive serial sample result after natural HPV infection.

Of the HPV vaccination study population, all 50 (100.0%) pre-vaccination samples were negative with the HPV16-L1 antibody rapid test, and 49/50 (98.0%) post-vaccination samples tested positive, and 1 (2.0%) negative. Finally, in the natural infection study population, all 26 (100.0%) pre-seroconversion samples were negative for the rapid test. Of the post-seroconversion samples, 9 samples (34.6%) tested positive for the HPV16-L1 antibody rapid test, and 17 (65.4%) negative (Table 2).

### Diagnostic accuracy of the HPV16-L1 antibody rapid test

Table 3 shows the diagnostic accuracy of the HPV16-L1 antibody rapid test for diagnosing HPV-OPC among patients with OPC. The results of the rapid test were compared with the tissue-based reference method of being positive for both HPV16 DNA and p16. The sensitivity of the rapid test for detecting HPV-OPC was 25.0% (95% CI: 13.6, 39.6), and specificity was 97.1% (95% CI: 85.1, 99.9). The NPV of the rapid test among patients with OPC was 48.6% (95% CI: 36.4, 60.8), while the PPV was 92.3% (95% CI: 64.0, 99.8). The proportion of correctly classified patients with OPC was 55.4% (95% CI: 44.1, 66.3).

**Table 3:** Diagnostic accuracy HPV16-L1 antibody rapid test (Prevo-Check®) for HPV-OPC among OPC patients.

Measure	Point estimate % (95% CI)
Sensitivity	25.0 (13.6, 39.6)
Specificity	97.1 (85.1, 99.9)
Positive predictive value	92.3 (64.0, 99.8)
Negative predictive value	48.6 (36.4, 60.8)
Correctly classified proportion	55.4 (44.1, 66.3)

Note: As reference method, HPV-OPC positivity was based on concurrent HPV16 DNA and p16^INK4a^ positivity, and otherwise was considered non-HPV-OPC.

Abbreviations: CI, confidence interval; HPV, human papillomavirus; HPV-OPC, HPV-driven OPC; OPC, oropharyngeal cancer.

### HPV16-L1 antibody titres

**Fig. 1:**
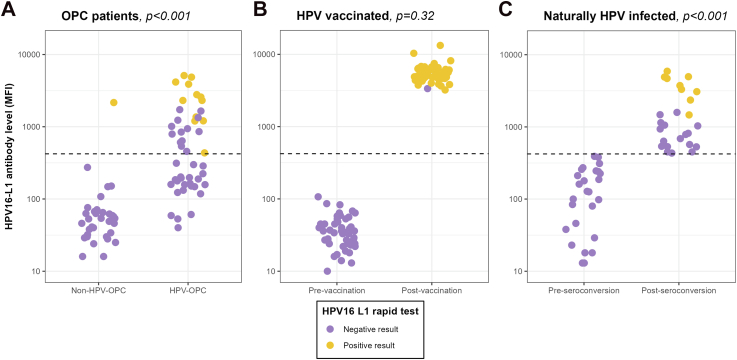
Comparison of HPV16-L1 antibody titres with HPV16-L1 antibody rapid test for oropharyngeal cancer patients (A), HPV vaccine recipients (B) and naturally HPV infected individuals (C). 1: HPV-driven was considered when patients with OPC were HPV16 DNA and p16 positive, otherwise patients with OPC were considered non-HPV-OPC. 2: Post-vaccination was after receiving 3 doses of Cervarix® and 1 month after receiving the third dose. All participants were negative for HPV DNA at baseline and 7 months later. 3: Seroconversion is based on last HPV16-L1 antibody negative and first HPV16-L1 antibody positive serial sample result after natural HPV infection. Note: *p*-values presented for the McNemar's test. A *p*-value <0.05 indicates there is a significant difference in the detection rates between the HPV16-L1 antibody rapid test and the multiplex serology assay. For the multiplex serology assay, a positivity cut-off was pre-defined at 422 MFI. Abbreviations: HPV-OPC, HPV-driven oropharyngeal cancer; non-HPV-OPC, non-HPV-driven oropharyngeal cancer.

Fig. 1 presents per study population, the HPV16-L1 antibody levels as defined according to the multiplex serology assay in relation to the HPV16-L1 antibody rapid-test results (Supplementary Table S1). Significant differences in HPV16-L1 positivity between the tests were observed among patients with OPC and among the naturally HPV-infected individuals (*p* < 0.001 for both study populations). The largest discordance for patients with OPC was observed among the HPV-OPC patients, where more HPV-driven OPC were positive according to multiplex serology (n = 26, 31.3%) than for the rapid test (n = 13, 15.7%) (Fig. 1A). For the naturally HPV infected individuals, all post-seroconversion individuals were positive according to multiplex serology, where only 34.6% was positive with the rapid test (Fig. 1C). However, for the HPV vaccine recipients, the McNemar's test did not find a significant difference in marginal positivity (*p* = 0.32) (Fig. 1B).

## Discussion

With the increasing incidence of HPV-OPC in recent years, early detection is very important for improving prognosis. In our study, we first assessed the diagnostic accuracy of the HPV16-L1 antibody rapid test (Prevo-Check®) as diagnostic tool for HPV-OPC among patients with OPC. The rapid test was able to identify 25.0% (95% CI: 13.6, 39.6) of the HPV-OPC patients in the study population of OPC patients. The test classified 97.1% (95% CI: 85.1, 99.9) of non-HPV-OPC patients correctly as HPV-negative. The PPV was 92.3% (95% CI: 64.0, 99.8), and the NPV was 48.6% (95% CI: 36.4, 60.8). However, the test also yielded positive results in individuals without HPV-OPC but with HPV16-L1 antibodies due to other factors, such as prior HPV vaccination or natural HPV infection. Among HPV vaccine recipients, 98.0% tested positive for HPV16-L1 antibodies with the rapid test, and 34.6% of individuals with a recent natural HPV infection tested positive as well.

Currently, only one study, by Weiland et al., has reported on the diagnostic accuracy of the Prevo-Check® rapid test for detecting HPV-OPC. That study combined several small study populations to assess diagnostic accuracy, and reported a sensitivity of 95.0% (95% CI: 77.2, 99.9) among 34 patients with OPC.^11^ This contrasts sharply with our observations, despite comparable characteristics of the study populations in terms of age, sex, and proportion of HPV-OPC. In our study, the manufacturer's instructions were strictly followed by trained personnel of the German National Reference Center for Papilloma- and Polyomaviruses blinded with regard to sample characteristics and clinical data. The test results were independently assessed by two members of the Reference Center and photo-documented. Whether lab personnel was also blinded in the study of Weiland et al., or how the interpretation was handled, was unfortunately not reported. Although the rapid test yields only a binary result (positive/negative), some degree of subjective interpretation in borderline cases cannot be excluded. In our study, this possibility was minimised by blinded, independent assessments; whether similar safeguards were applied in the study by Weiland et al. was not reported. The definition for HPV-OPC used in both study populations was identical, and cancer stage was not associated with the rapid test outcome.^11^ Therefore, the sensitivity difference due to other reasons remains unexplained. Although unlikely, minor variations between test lots could theoretically contribute to such differences. The test lots of both studies could not be compared directly to exclude this potential cause.

The specificity of the HPV16-L1 antibody rapid test reported by Weiland et al. was over 99%. However, this specificity was estimated using a control group of 1064 healthy C-reactive protein-negative individuals,^11^ which is a different category of controls than in our study. Our study assessed all the diagnostic accuracy measures, including specificity, exclusively within a population of patients diagnosed with OPC, instead of using separate study groups for different metrics. Therefore, a direct comparison of specificity estimates between the two studies would not be appropriate.

Nonetheless, because Weiland et al. provided individual-level rapid test and HPV-status data for all 34 patients with OPC, it is possible to re-calculate specificity using the same method we applied. In our study, the specificity of the rapid test was 97.1% (95% CI: 85.1, 99.9) among patients with OPC. Based on this approach, the specificity of Weiland et al. among their patients with OPC would be 76.9% (95% CI: 49.7, 91.8), which is lower than the specificity observed in our analysis.

Our study observed a PPV of 92.3% (95% CI: 64.0, 99.8), and a NPV of 48.6% (95% CI: 36.4, 60.8). The study of Weiland et al. observed a PPV of 45.6% and a NPV of 99.9% (confidence intervals not provided).^11^ Similar to the sensitivity and specificity, the observations of our study and the study of Weiland et al. have a substantial discrepancy. Since the PPV and NPV in their study were calculated using the same OPC patient group as for sensitivity and, given that the study population characteristics of the two studies are comparable, the reason for this discrepancy remains unclear.

As part of our evaluation of the HPV16-L1 antibody rapid test, we included two study populations that did not have HPV-OPC, but were HPV16-L1 positive due to other factors (i.e., HPV vaccine recipients and naturally HPV16 infected individuals). In both these study populations, a considerable number of individuals tested positive in the rapid test. Given their young age and inclusion criteria, which required negative baseline results for both HPV DNA and serology, it is highly unlikely that these individuals had HPV-OPC. Therefore, we consider the positive results of the HPV16-L1 antibody rapid test as false-positives regarding the detection of HPV-OPC in these study populations. Recently, the manual for the rapid test was updated, now advising against its use within the first six years after HPV vaccination, as vaccine-induced antibodies may cause false positives. However, no evidence suggests that antibody levels decline after six years. On the contrary, many studies showed that antibody levels remain stable well beyond this period,^23^, ^24^, ^25^, ^26^, ^27^ even after a single vaccine dose.^25^^,^^27^ Whether the level of antibodies after one dose would be sufficient to result in false-positives has to be investigated in future research. The high proportion of the post-vaccine individuals and a part of the recently HPV-infected that tested positive on the rapid test, suggest that the test accurately detects L1 antibodies, but these alone have shown to be of limited value for identifying HPV-OPC. Previous studies indicate that antibodies against other HPV16 proteins, such as E6 or E7 oncoproteins, may elicit stronger and more specific responses in patients with OPC, and could therefore serve as more reliable serologic markers for early detection.^28^^,^^29^

Several limitations should be acknowledged. First, our diagnostic accuracy assessment was conducted among patients already diagnosed with OPC, rather than in a general screening population. Ideally, a rapid test would be evaluated as a screening tool for detecting HPV-OPC in the general population. However, given the rarity of OPC, such a study would require an extremely large sample size. Therefore, most research on HPV-related OPC, including our study, has been conducted exclusively on samples of patients already diagnosed with OPC rather than using pre-diagnostic samples. Additionally, for a rare disease like HPV-OPC, the specificity would have to be near 100% in order to be useful as a screening tool in the general public, which is challenging to achieve. Therefore, evaluating the rapid test as a diagnostic tool in a clinical setting is more appropriate.

Second, all HPV-OPC included in this study were HPV16-driven, as the HPV16-L1 antibody test targets this genotype. As roughly 90% of HPV-OPC are attributed to HPV16,^30^ the test and consequently our analyses may miss the remaining 10% of HPV-OPC cases caused by other HPV genotypes. Yet, the rapid test is based on HPV16-L1 antibodies, making the definition of HPV-OPC as used in the study the most appropriate for evaluating the performance of the rapid test.

Third, we included only recently HPV infected participants in our analyses that had a seropositive result, despite the fact that only a proportion of HPV infections will lead to seroconversion.^31^ This inclusion criterion was based on two considerations. As HPV DNA detection methods are typically highly sensitive, relying solely on DNA test results could lead to false positives. For example, viral DNA fragments may be detected rather than active infections. By selecting participants with both positive HPV16-DNA results followed by positive serology results, we increased the likelihood of identifying true infections. The second consideration was that this study is intended for proof-of-concept, rather than an observational study. Future research could expand on this by evaluating the rapid test in a study population of naturally HPV16 infected individuals, irrespective of seroconversion.

Finally, both vaccinated and newly HPV-infected participants in this study were younger than patients with OPC, and were predominantly female. In practice, screening or diagnostic tools for OPC would likely be used primarily in older male individuals due to their increased risk for OPC.^32^ This may limit the generalisability of our findings to the population most likely to undergo HPV-OPC screening. Nevertheless, these cohorts are informative for illustrating potential sources of false positives in individuals with HPV16-L1 antibodies from vaccination or natural infection and were selected to demonstrate proof-of-concept. Additionally, HPV antibodies levels remain high over many years after HPV vaccination, even after receiving a single dose,^25^^,^^27^ suggesting it is highly plausible that vaccine recipients will remain seropositive into the age range associated with the highest risk for HPV-OPC. This seropositivity due to vaccination may result in false-positives when using the HPV16-L1 antibody rapid test for detecting HPV-OPC. Additionally, HPV infections can be acquired at all ages, including older ages. Notably, the prevalence of oral and male genital HPV infection does not decrease with age, and is much more common than HPV-OPC.^33^^,^^34^ Consequently, seroconversion following HPV infection at any anatomical site may occur in the age range associated with the highest risk for HPV-OPC, increasing the likelihood of false-positive results.

In conclusion, serological assays based on HPV16-L1 antibody rapid tests cannot reliably distinguish various sources of HPV16-L1 antibodies as they generate positive results for HPV vaccinees, individuals after natural HPV infection, as well as a low proportion of HPV-OPC cases at diagnosis. The very low sensitivity, low negative predictive value, and false-positives among certain groups, suggests that the HPV16-L1 antibody rapid test may not be suitable as a screening tool for the general public. Similarly, another test based on HPV16-L1 (CancerCheck®) for detecting cervical cancer and its precursors demonstrated suboptimal performance, as all vaccinated controls tested positive.^35^ Therefore, despite efforts to optimise assays based on L1 antibodies, tests based on this antibody may not be appropriate for screening and early detection.

However, continued research on HPV-OPC early diagnosis and screening remains very important. For example, the progression from oral HPV infections to HPV-OPC is still not well understood. Further research into the progression may lead to new biomarkers for HPV-OPC which are detectable short before onset of the disease. Additionally, identifying high risk populations could increase the feasibility of screening for HPV-OPC. Although the HPV16-L1 antibody rapid test may not be appropriate for screening and early detection, other tools may be. Previous research has been conducted on oral rinse HPV-DNA testing, antibodies against HPV oncoproteins, circulating tumour HPV DNA, and other potential biomarkers. Continued research in this area is urgently needed for improving early detection of HPV-OPC.

## Contributors

Conceptualisation, T.W., N.B., M.Z., E.L.F., M.L., J.P.K., U.W.; Methodology, T.W., N.B., J.M.A.K., J.P.K., S.S., U.W., P.G., H.K.; Investigation, S.S., U.W., N.B.; Data curation, J.M.A.K., N.B.; Formal analyses, J.M.A.K., N.B.; Writing original draft, J.M.A.K.; Writing—review and editing, T.W., J.A.B., U.W., M.Z., P.G., S.S., H.K., E.L.F., M.L., A.D., S.L., J.P.K., J.M.A.K.; Validation, J.M.A.K., N.B; Visualisation, J.M.A.K.; Supervision, T.W., U.W., J.A.B.; Funding acquisition, T.W., J.P.K., U.W.

All authors read and approved the final manuscript.

## Data sharing statement

The data that support the findings of this study are available from the corresponding author upon reasonable request.

## Declaration of interests

T.W. serves on advisory boards for MSD. J.A.B. is principal investigator of an investigator-initiated study from GSK. E.L.F. received grants from the Canadian Institutes of Health Research, grants and fees from Merk, has a patent related to the discovery “DNA methylation markers for early detection of cervical cancer” and has an editorial role at Oxford University Press. S.L. served in the advisory board for MSD, Bristol Myers Squibb and Böhringer Ingelheim, did consulting for MSD, GSK, Böhringer Ingelheim, and Bristol Myers Squibb and has a patent pending for “Peptides for antigen-specific immunotherapy of head and neck cancer or other cancers (EP2023/071,063)”. J.P.K. has received grants from MSD and honoraria for lectures from MSD, BMS and Merck. M.Z. holds a patent related to the discovery “DNA methylation markers for early detection of cervical cancer”, registered at the Office of Innovation and Partnerships, McGill University, Montreal, Quebec, Canada (October 2018). A.D. received grants from MSD, honoraria for lectures from MSD, Merck serono, Nanobiotix and Regeneron, and served on advisory boards of MSD, GSK, Bristol Myers Squibb and Regeneron, and has a leadership role in IAG-KHT. All other authors report no potential conflicts.
