# Supplementary material for: Evaluation of an HPV16-L1 antibody rapid test for oropharyngeal cancer diagnosis: diagnostic accuracy and challenges in real-world settings
Source: eBioMedicine. 2025 Dec 11;123:106057. doi: 10.1016/j.ebiom.2025.106057 (PMC12902241; doi:10.1016/j.ebiom.2025.106057)
Supplement: Supplementary Table S1 [file mmc1.docx]

*Supplement to*

**Evaluation of an HPV16-L1 Antibody Rapid Test for Oropharyngeal Cancer Diagnosis: Diagnostic Accuracy and Challenges in Real-World Settings**

| **Supplementary Table 1:** Comparison of HPV16-L1 antibody titres with HPV16-L1 antibody rapid test for oropharyngeal cancer patients, HPV vaccine recipients and naturally HPV infected individuals. | | |
| --- | --- | --- |
| **Study population 1: OPC patients^1^** | | |
|  | **HPV16-L1 antibody rapid test (Prevo-Check®)** | |
| **Multiplex serology assay** | **Negative** | **Positive** |
| **Negative** | 57 | 0 |
| **Positive** | 13 | 13 |
| **Study population 2: HPV vaccine recipients^2^** | | |
|  | **HPV16-L1 antibody rapid test (Prevo-Check®)** | |
| **Multiplex serology assay** | **Negative** | **Positive** |
| **Negative** | 50 | 0 |
| **Positive** | 1 | 49 |
| **Study population 3: Naturally HPV infected individuals^3^** | | |
|  | **HPV16-L1 antibody rapid test (Prevo-Check®)** | |
| **Multiplex serology assay** | **Negative** | **Positive** |
| **Negative** | 26 | 0 |
| **Positive** | 17 | 9 |
|  | | |
| *1: HPV-OPC positivity was based on concurrent HPV16 DNA and p16^INK4a^ positivity, and otherwise was considered non-HPV-OPC.* | | |
| *2: Post-vaccination was after receiving 3 doses of Cervarix® and 1 month after receiving the third dose. All vaccinated 16- and 17-year-old females were negative for HPV DNA at baseline and 7 months later.* | | |
| *3: Seroconversion is based on last HPV16-L1 antibody negative and first HPV16-L1 antibody positive serial sample result after natural HPV infection.*  *4: Positivity for Multiplex serology defined by the pre-specified cut-off of 422 MFI* | | |
| *Abbreviations: HPV-OPC, HPV-associated oropharyngeal cancer; non-HPV-OPC, non-HPV-associated oropharyngeal cancer* | | |
